# A Rare Case of Juvenile Idiopathic Arthritis following a Ruptured Baker's Cyst in a Toddler

**DOI:** 10.1155/2020/1601348

**Published:** 2020-04-06

**Authors:** Deniz Üstüner, Fatma Asaid, Muhammad Pervaiz, Godwin Oligbu

**Affiliations:** Department of Paediatrics, Dr Gray's Hospital, NHS Grampian, Aberdeen, UK

## Abstract

A Baker's cyst is usually an incidental finding in adults being investigated for a joint arthropathy, and its rupture preceding the diagnosis of juvenile idiopathic arthritis (JIA) is rare in children. Here, we describe a case of a 4-year-old girl who presented to the Emergency Department with right calf pain, swelling, and no preceding history of trauma. MRI confirmed a ruptured Baker's cyst with inflammatory arthropathy alongside an extensive synovial proliferation throughout the knee joint with large joint effusions and associated soft tissue oedema tracking superiorly and inferiorly along the medial head of gastrocnemius and anteriorly along the tibia. Further investigations revealed bilateral uveitis consistent with a diagnosis of juvenile idiopathic arthritis.

## 1. Background

A Baker's cyst (or popliteal cyst) is a mass located in the popliteal area, filled with fluid [1]. An extension of this is the synovial popliteal cyst: a communication of the gastrocnemius and semimembranous bursa with the posterior joint capsule [2]. A rupture within this communication causes fluid to drain down, resulting in calf swelling. In adults, the condition usually manifests with previous intra-articular conditions [3], whereas children may have no coexisting or previous knee pathology [4]. Other causes of chronic knee such as tendon pathologies, e.g., semimembranous tendinopathy, can present with a similar clinical picture [5].

Clinical examination alone is a poor determinant for diagnosis of a Baker's cyst, with ultrasonography (US) and magnetic resonance imaging (MRI) usually more reliable imaging modalities for identification of a popliteal swelling in these patients. The prevalence of a Baker's cyst in children on MRI on routine imaging investigating for other joint pathology is 6.3% [6], whereas in adults this can be up to 19% [7]. The most common complication of a Baker's cyst in adult population is rupture, which can be up to 50% of a presenting complicated Baker's cyst, with as many as 80% being asymptomatic [8].

However, there are very few reported cases of ruptured Baker's cysts presenting in children with majority of them already diagnosed with juvenile idiopathic arthritis (JIA): a set of inflammatory disorders without a fully identified aetiology. In the UK, approximately 1000–1500 diagnoses are being made in children every year [9]. Children typically present with pain in one or more joints for weeks and months, and if left untreated can lead to ongoing pain and significant disability [10]. Calf pain and swelling as clinical presentation for a ruptured Baker's cyst in the setting of a JIA is rare. In the literature, there are very few cases of paediatric patients with a first presentation of knee swelling and pain in the context of JIA [11], whereas in the adult population it is more likely that a popliteal cyst will present with arthritic conditions or internal derangement within the knee joint. Here, we describe a toddler who presented with a right calf swelling following a ruptured Baker's cyst, demonstrating the diagnostic challenges and highlighting a high index of suspicion in this age group.

## 2. Case Presentation

A 4-year-old girl presented to the Emergency Department (A&E) with a 3 week history of right calf swelling after her usual, nontraumatic dancing. The swelling had worsened over 48 hours. There was no history of trauma to the area, no erythema, and she had been apyrexial. She regularly went dancing and participated in acrobatics. There was no family history of clotting disorder or joint arthropathy.

On examination, her weight was just above the 75th centile at 20 kg, with a BMI of 17.9. She was unable to fully extend her right leg or weight bear due to her calf swelling. Her right calf measured 28.6 cm compared to the left calf of 26.4 cm. She was able to perform a straight leg raise test with all ligaments intact, no demonstrable joint effusion, and no issues with joints above and below.

Three weeks prior to her presentation, she was seen in the A&E with pain and swelling in her right calf but was able to weight bear at this point. No imaging was conducted then, and she was discharged home with the diagnosis of soft tissue injury. On further questioning, the patient's mother revealed that she had been experiencing leg pain for over 6 months without noticeable leg swelling. Family history consisted of Crohn's and coeliac disease on the maternal side.

Her initial X-rays of her right knee, tibia, and fibula on presentation showed no bony injury and unremarkable soft tissue swelling when compared to her previous left knee/tibia/fibula X-rays. A urine dipstick was negative to blood, protein, and cells with a normal specific gravity. Blood results showed an erythrocyte sedimentation rate (ESR) of 26 mm, C-reactive protein (CRP) < 4 mg/L, and a normal white cell and neutrophil count. Her prothrombin time was slightly increased at 13.4; however, the extended clotting profile for factor VII, factor VIII, factor XIII, and von Willebrand factor were all normal. The complement levels (C3 and C4) were within normal ranges, including the anti-nuclear antibodies (ANA), and rheumatoid factors were also negative. An ultrasound (US) of the affected joint showed a 43 × 10 mm collection within gastrocnemius with a possible haematoma which raised the suspicion of a venous thromobosis. Another differential was the possibility of a fast growing tissue sarcoma, but her lactate dehydrogenase was unremarkable.

**Figure 1:**
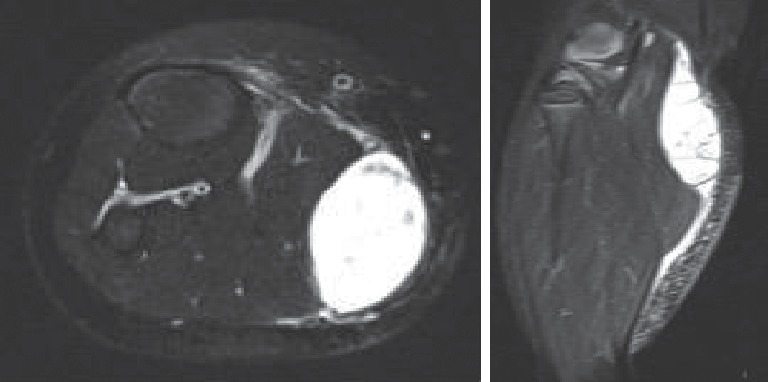
MRI of Baker's cyst of the case with partial rupture in the superfical posterior compartment.

She was commenced on conservative management and discharged home on nonsteroidal anti-inflammatory medication pending her MRI investigations. Three weeks later, her calves were unchanged in size and she had full and painless range of movement in both knee joints, but an effusion was still evident in her right knee with a firm swelling in the posteromedial aspect of the proximal calf. The MRI showed an inflammatory arthropathy with a large Baker's cyst (4.3 × 2.2 × 9.4 cm). There was extensive synovial proliferation throughout the knee joint with large joint effusions. There was also a clear communication within the Baker's cyst, with numerous septations within the encapsulated lesion and associated soft tissue oedema tracking superiorly and inferiorly along the medial head of gastrocnemius and anteriorly along the tibia (Figure 1).

She was seen in our rheumatology clinic and was diagnosed with a juvenile idiopathic arthritis, a monoarthritis of her right knee on a background of a ruptured Baker's cyst. Ophthalmology review showed bilateral anterior uveitis, and she was commenced on topical eye drops. In addition, a short course of oral steroids was prescribed, and an intraarticular steroid injection was given into her right knee joint (triamcinolone hexacetonide). This was continued with a weekly subcutaneous methotrexate with a referral to the physiotherapy.

Since her initial acute presentation, the pain within the patient's right knee had improved significantly and the calf swelling had subsided.

## 3. Discussion

This case highlights the atypical presentation of JIA, and the clinical manifestations are highly variable in the presence of a ruptured Baker's cyst. Baker's cysts are common cystic lesions in the knee joint, which are prevalent among the adult population but rare in children. They are usually asymptomatic in the majority of cases, and only identified incidentally during evaluation for knee arthropathy. An isolated rupture of a Baker's cyst is rarely reported in children, with the first published case in a 15-year-old teenager [12]. Similar to this case, although much younger, this child presented with calf swelling and a diagnosis of venous thrombosis was initially entertained. This is a known differential for calf swelling, and it is not surprising as the synovial fluid could leak from the joint space and cause swelling in the calf muscles. In addition, the initial findings, on the US of haematoma contributed to the diagnostic challenge and dilemma.

This toddler was commenced initially on conservative management with no improvement in her clinical condition, and by the third week, the MRI of the right knee was suggestive of inflammatory arthropathy associated with a ruptured large Baker's cyst. Though, US is generally the first line imaging of choice for investigating and diagnosing a Baker's cyst, with a very good yield. However, in children with a complicated history, an MRI may be required to demonstrate atypical locations, possible extension to the tibio-fibular joint-gastrocnemius and biceps muscles, or any other comorbidities [2].

Calf pain and swelling as clinical presentation for a ruptured Baker's cyst or JIA is rare. In the literature, there are very few cases of paediatric patients with a first presentation of knee swelling and pain in the context of JIA [10], whereas in the adult population it is more likely that a popliteal cyst will present with arthritic conditions or internal derangement within the knee joint. In one study consisting of 44 patients aged 2–15 years over 8 years with an average follow-up of 32.1 months who presented with popliteal cysts, only one was found to have a diagnosis of Ehlers–Danlos Syndrome and no other arthropathies were diagnosed, with spontaneous resolution of the cyst [13]. However, in another case series of 16 paediatric patients aged 4 to 16 years with a clinical diagnosis of a Baker's cyst, 3 were found to have JIA [4]. These 3 children were already diagnosed with JIA before the development of the Baker's cyst, and none of the cases had ruptured, suggesting that even in children with prior inflammatory arthropathy, the occurrence of a ruptured Baker's cyst is uncommon. To our understanding, this is the first case of JIA reported in a toddler following a ruptured Baker's cyst.

Nonetheless, the majority of popliteal cysts in children are benign with no associated joint arthropathies, and are therefore managed conservatively. In one follow-up of MRI imaging in one paediatric cohort, 85% cysts (17/20) disappeared or were significantly reduced in size without surgical intervention [14]. This case is not without its limitation. The initial US did not explore in detail the right knee joint and structures. In addition, we have reported only one case, which is generally of low evidence and this needs to be further verified in a population study. However, in this case, resolution of joint pain and swelling only occurred after initiation of a steroid injection, suggesting that it is critical to evaluate the underlying cause to the ruptured Baker's cyst or the coincidental presence, in order to institute the appropriate management expeditiously.

## 4. Conclusions

Unlike adults, Baker's cyst is usually an incidental finding in children which is managed conservatively, and the majority would have no other associated comorbidity. We recommend that clinicians consider a possible joint inflammatory arthropathy in children presenting with a ruptured Baker's cyst as early diagnosis and treatment could reduce morbidity associated with this inflammatory arthropathy.
